# Supplementary material for: Urban Bird Community Assembly Mechanisms and Driving Factors in University Campuses in Nanjing, China
Source: Animals (Basel). 2023 Feb 15;13(4):673. doi: 10.3390/ani13040673 (PMC9952131; doi:10.3390/ani13040673)
Supplement: Supplementary file 1 [file animals-13-00673-s001.zip › animals-2129567-supplementary.pdf]

Supplementary Materials

Table S1. The best prediction models of functional diversity (FDMPD, FDMNTD) and phylogenetic diversity (PDMPD, PDMNTD) showing in the order of increasing AICc (Akaike Information Criterion for small sample size), △AICc and weight (Akaike weights) based on the multiple linear regression models. df = degrees of freedom, logLik = log likelihood. adj. R^2 = adjusted R^2. For abbreviations see Table 1.

Time	Response variable	Models	df	logLik	AICc	ΔAICc	weight	adj.R^2
year	FD	water	4	-6.66	27.03	0	0.38	0.00
	FDMPD	building+water	5	-7.12	34.23	0	0.71	0.24
	FDMNTD	water	4	-4.32	22.36	0	0.31	0.02
	PD	grass+water	5	-8.93	37.87	0	0.8	0.36
	PDMPD	water	4	-6.27	26.26	0	0.31	-0.22
	PDMNTD	water	4	-5.07	23.85	0	0.25	-0.04
spring	FD	grass+water	5	-1.29	22.59	0	0.94	0.93
	FDMPD	water	4	-7.77	29.25	0	0.7	0.02
	FDMNTD	building+water	5	-6.5	33.01	0	0.52	0.61
	PD	water	4	-7.44	28.6	0	0.37	0.33
	PDMPD	water	4	-10.59	34.89	0	0.37	-0.09
	PDMNTD	water	4	-9.05	31.82	0	0.43	0.00
summer	FD	building+water	5	-7.37	34.74	0	0.43	0.36
	FDMPD	water	4	-9.42	32.55	0	0.39	0.25
	FDMNTD	water	4	-6.86	27.44	0	0.63	0.69
	PD	building+grass+water	6	1.66	25.47	0	0.55	0.92
	PDMPD	grass+water	5	-5.46	30.92	0	0.29	0.16
	PDMNTD	grass+water	5	-5.51	31.01	0	0.29	0.06
autumn	FD	water	4	-12.12	37.95	0	0.43	0.42
	FDMPD	building+water	5	-11.25	42.5	0	0.41	0.18
	FDMNTD	water	4	-11.82	37.35	0	0.41	0.46
	PD	grass+water	5	-7.51	35.01	0	0.52	0.56
	PDMPD	water	4	-15.57	44.85	0	0.34	0.14
	PDMNTD	water	4	-15.43	44.57	0	0.37	0.02
winter	FD	grass+water	5	-6.02	32.04	0	0.38	0.43
	FDMPD	water	4	-13.77	41.25	0	0.32	0.07
	FDMNTD	grass+water	5	-9.23	38.46	0	0.36	0.49
	PD	building+water	5	-6.62	33.25	0	0.48	0.37
	PDMPD	building+water	5	-9.62	39.24	0	0.52	0.18
	PDMNTD	building+water	5	-4.07	28.15	0	0.43	0.05

Table S2. List of birds in 12 campuses of Nanjing, three animals protection that means beneficial, has important economic value and scientific research value, II is the second-class protected animal.

Order	Family	Species	Resident	geographic area	protection class
Podicipediformes	Podicipedidae	Tachybaptus ruficollis	Resident	Widespread species	Three animals protection
Ciconiiformes	Ardeidae	Egretta garzetta	Summer migrant	Oriental Realm	Three animals protection
		Ardeola bacchus	Summer migrant	Oriental Realm	Three animals protection
		Nycticorax nycticorax	Resident	Widespread species	Three animals protection
		Bubulcus ibis	Summer migrant	Oriental Realm	Three animals protection
		Butorides striata	Summer migrant	Oriental Realm	Three animals protection
Gruiformes	Rallidae	Gallinula chloropus	Resident	Widespread species	Three animals protection
Columbiformes	Columbidae	Streptopelia orientalis	Resident	Widespread species	Three animals protection
		Spilopelia chinensis	Resident	Oriental Realm	Three animals protection
		Columba livia	Resident	Widespread species	Three animals protection
Passeriformes	Pycnonotidae	Pycnonotus sinensis	Resident	Widespread species	Three animals protection
		Spizixos semitorques	Resident	Oriental Realm	Three animals protection
		Pycnonotus jocosus	Winter migrant	Oriental Realm	Three animals protection
	Motacillidae	Motacilla alba	Resident	Palearctic realm	Three animals protection
		Motacilla cinerea	Passenger	Palearctic realm	Three animals protection
		Anthus hodgsoni	Winter migrant	Palearctic realm	Three animals protection
	Laniidae	Lanius schach	Resident	Oriental Realm	Three animals protection
		Lanius cristatus	Summer migrant	Palearctic realm	Three animals protection
	Corvidae	Cyanopica cyanus	Resident	Palearctic realm	Three animals protection
		Pica pica	Resident	Widespread species	Three animals protection
		Urocissa erythrorhyncha	Resident	Oriental Realm	Three animals protection
		Dendrocitta formosae	Resident	Oriental Realm	Three animals protection
	Turdidae	Turdus merula	Resident	Oriental Realm	Three animals protection

	Turdus hortulorum	Winter migrant	Palearctic realm	Three animals protection
Timaliidae	Garrulax perspicillatus	Resident	Oriental Realm	Three animals protection
Ploceidae	Passer montanus	Resident	Widespread species	Three animals protection
	Lonchura striata	Resident	Oriental Realm	Three animals protection
Passeridae	Eophona migratoria	Resident	Palearctic realm	Three animals protection
	Carduelis sinica	Resident	Widespread species	Three animals protection
	Fringilla montifringilla	Winter migrant	Palearctic realm	Three animals protection
Dicruridae	Dicrurus macrocercus	Resident	Widespread species	Three animals protection
Paradoxornithidae	Paradoxornis webbianus	Resident	Oriental Realm	Three animals protection
Paridae	Parus minor	Resident	Widespread species	Three animals protection
	Aegithalos caudatus	Resident	Palearctic realm	Three animals protection
	Aegithalos concinnus	Resident	Oriental Realm	Three animals protection
	Parus venustulus	Winter migrant	Oriental Realm	Three animals protection
Sturnidae	Acridotheres cristatellus	Resident	Oriental Realm	Three animals protection
	Sturnus sericeus	Resident	Oriental Realm	Three animals protection
	Sturnus cineraceus	Resident	Palearctic realm	Three animals protection
	Sturnus nigricollis	Winter migrant	Oriental Realm	Three animals protection
Muscicapidae	Copsychus saularis	Resident	Oriental Realm	Three animals protection
	Phoenicurus auroreus	Winter migrant	Palearctic realm	Three animals protection
	Tarsiger cyanurus	Winter migrant	Palearctic realm	Three animals protection
Sylviidae	Phylloscopus proregulus	Winter migrant	Palearctic realm	Three animals protection
	Phylloscopus inornatus	Winter migrant	Palearctic realm	Three animals protection
	Horornis fortipes	Resident	Oriental Realm	Three animals protection
Hirundinidae	Cecropis daurica	Summer migrant	Widespread species	Three animals protection
	Hirundo rustica	Summer migrant	Widespread species	Three animals protection
Campephagidae	Pericrocotus cantonensis	Passenger	Widespread species	Three animals protection

	Zosteropidae	Zosterops japonicus	Summer migrant	Oriental Realm	Three animals protection
Piciformes	Picidae	Dendrocopos canicapillus	Resident	Oriental Realm	Three animals protection
		Picumnus innominatus	Resident	Oriental Realm	Three animals protection
		Dendrocopos major	Resident	Oriental Realm	Three animals protection
		Picus canus	Resident	Oriental Realm	Three animals protection
Falconiformes	Accipitridae	Milvus migrans lineatus	Resident	Widespread species	II
Coraciiformes	Alcedinidae	Alcedo atthis	Resident	Widespread species	Three animals protection
		Ceryle rudis	Resident	Widespread species	Three animals protection
	Coraciidae	Eurystomus orientalis	Passenger	Oriental Realm	Three animals protection
Charadriiformes	Charadriidae	Vanellus cinereus	Winter migrant	Oriental Realm	Three animals protection
	Scolopacidae	Tringa ochropus	Winter migrant	Palearctic realm	Three animals protection
Cuculiformes	Cuculidae	Eudynamys scolopaceus	Resident	Oriental Realm	Three animals protection
		Hierococcyx sparverioides	Summer migrant	Oriental Realm	Three animals protection
		Cuculus micropterus	Summer migrant	Oriental Realm	Three animals protection
Anseriformes	Anatidae	Anas poecilorhyncha	Winter migrant	Palearctic realm	Three animals protection
Galliformes	Phasianidae	Bambusicola thoracica	Resident	Widespread species	Three animals protection
		Phasianus colchicus	Resident	Widespread species	Three animals protection
Bucerotiformes	Upupidae	Upupa epops	Resident	Widespread species	Three animals protection

Table S3. Campus bird survey transects

Campus	Length of the first transect (km)	Length of the second transect (km)	Total length of transects (km)
NFU1	0.80	1.43	2.23
NU1	0.54	0.54	1.08
NNU1	0.51	0.52	1.03
NAAU1	0.98	0.89	1.87
NAU1	0.93	0.91	1.84
SEU1	0.80	0.88	1.68
NU2	1.68	1.75	3.43
NNU2	0.94	0.82	1.76
NCIT2	0.77	0.64	1.41
NFEU2	1.07	1.22	2.29
NCMU2	1.16	1.21	2.37
NPTU2	1.03	0.94	1.97

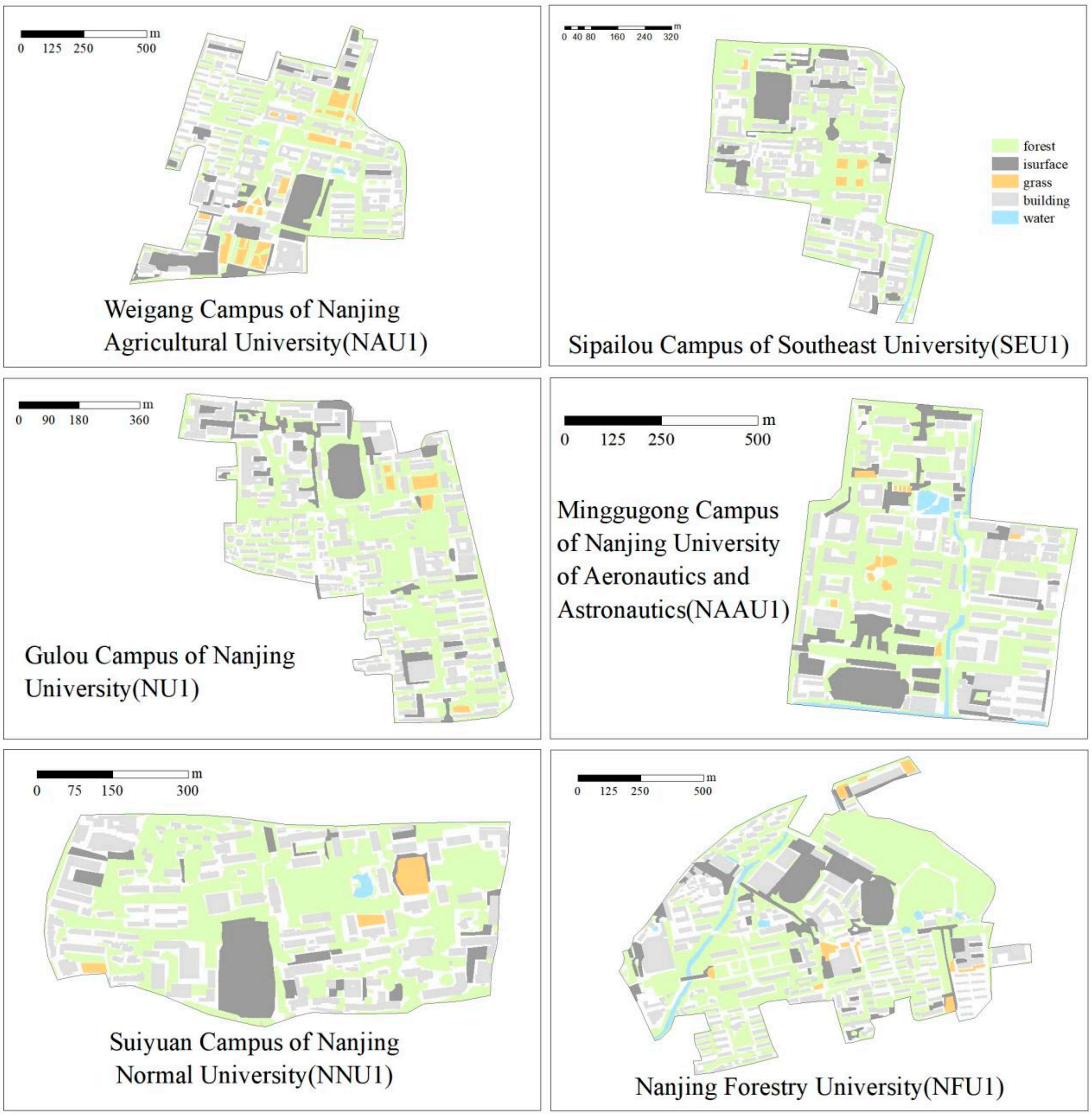

Figure S1. Classification of land use types of six universities in the downtown area.

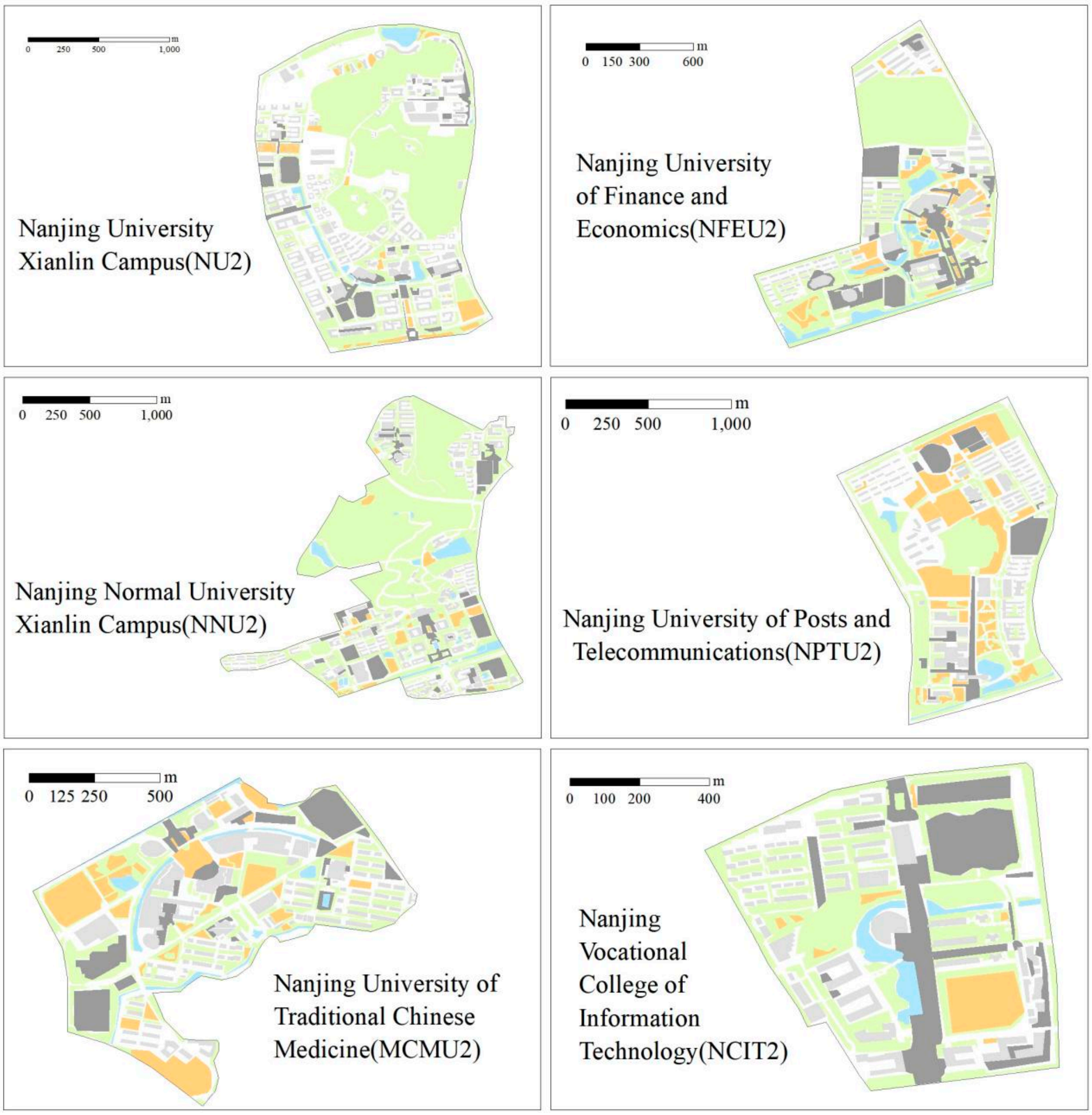

Figure S2. Classification of land use types of six universities in the newly developed area.
